# Multicomponent Gold-Linked Glycoconjugate Vaccine Elicits Antigen-Specific Humoral and Mixed T_H_1-T_H_17 Immunity, Correlated with Increased Protection against Burkholderia pseudomallei

**DOI:** 10.1128/mBio.01227-21

**Published:** 2021-06-29

**Authors:** Daniel Tapia, Javier I. Sanchez-Villamil, Heather L. Stevenson, Alfredo G. Torres

**Affiliations:** a Department of Microbiology and Immunology, University of Texas Medical Branch, Galveston, Texas, USA; b Department of Pathology, University of Texas Medical Branch, Galveston, Texas, USA; University of Toledo College of Medicine; Cornell University

**Keywords:** *Burkholderia pseudomallei*, melioidosis, gold nanoparticles, vaccine

## Abstract

Burkholderia pseudomallei is the causative agent of melioidosis, a fatal disease with a high mortality rate. The intrinsic resistance to commonly used antibiotics combined with the complex bacterial life cycle has hampered the development of preventive and therapeutic interventions and vaccines. Furthermore, the need of humoral and cell-mediated immunity in protection against B. pseudomallei has complicated the development of effective vaccines. Antigen delivery vaccine platforms that promote humoral and cellular responses while maintaining a safe profile are a roadblock to developing subunit vaccines against intracellular pathogens. Gold nanoparticles (AuNPs) were used for the delivery of multicomponent antigens with the goal of inducing vaccine-mediated immunity, promoting protection against melioidosis disease. Different nanoglycoconjugates using predicted immunogenic protein candidates, Hcp1, FlgL, OpcP, OpcP1, OmpW, and hemagglutinin, were covalently coupled to AuNPs, together with the lipopolysaccharide (LPS) from Burkholderia thailandensis, which acted as an additional antigen. Animals immunized with individually coupled (AuNP-protein-LPS) formulations containing OpcP or OpcP1, together with CpG as an adjuvant, showed a significant increase in protection, whereas a nanovaccine combination (AuNP-Combo2-LPS) showed significant and complete protection against a lethal intranasal B. pseudomallei challenge. Animals immunized with AuNP-Combo2-LPS showed robust humoral antigen-specific (IgG and IgA) responses with higher IgG2c titer, indicating a T_H_1-skewed response and promotion of macrophage uptake. In addition, immunization with the nanovaccine combination resulted in a mixed antigen-specific T_H_1-T_H_17 cytokine profile after immunization. This study provides the basis for an elegant and refined multicomponent glycoconjugate vaccine formulation capable of eliciting both humoral and cell-mediated responses against lethal B. pseudomallei challenge.

## INTRODUCTION

Burkholderia pseudomallei is an intracellular pathogen with a complex life cycle and the causative agent of melioidosis, a human disease associated with a high fatality rate (1). Melioidosis can be acquired by several routes, including inhalation, ingestion, or percutaneous inoculation (1, 2). A broad spectrum of complications can result from inoculation by any infection route, resulting in mortality rates exceeding 50% in some regions (1, 3). Prediction modeling highlighted the global burden of melioidosis to comprise 165,000 new cases each year, of which half are fatal (3). Together with the intrinsic resistance of B. pseudomallei to first-line antibiotics and prolonged treatment regimen for melioidosis, prompt antimicrobial therapy optimal for B. pseudomallei is crucial to control the infection (1, 4). However, melioidosis in regions of endemicity is associated with the highest mortality rates, attributed to failure of early detection and limited health care infrastructures, reinforcing the need for other effective preventive countermeasures.

The intricate host-pathogen interplay complicates the development of vaccines that are safe for use in susceptible subjects while inducing protective immune responses. Individuals who survive B. pseudomallei infection develop strong B. pseudomallei-specific humoral and cell-mediated responses, particularly evoking an immune response skewed toward the activation of T_H_1 cells, which is associated with gamma interferon (IFN-γ) production (5–10). In particular, human convalescent patients have higher antibody levels against lipopolysaccharide (LPS) and increased levels of CD4^+^ and CD8^+^ T cells (6, 10–12). Individuals who succumb to infection have decreased levels of T cells and reduced IFN-γ and interleukin-17 (IL-17) expression (6, 10, 12–15). While the detoxified form of LPS in several Gram-negative pathogens can act as an immune modulator or vaccine antigen, few vaccine strategies have exploited codelivery to target immune responses against different antigens. Therefore, a vaccine utilizing the *Burkholderia* LPS is predicted to provide increased protection against lethality. Further, the pool of protective antigens, together with the dearth in vaccine delivery platforms, which can be associated with the induction of robust antibody and cell-mediated immunity, has hindered the development of fully protective B. pseudomallei subunit vaccines (5). Another challenge is that sterilizing protection against inhalational exposure, which is associated with the highest mortality, remains a challenge in small-animal models. The induction of mucosa-specific immunity via nasal vaccination, although appealing, has been hindered by factors such as inefficient antigen uptake, size-restricted permeability, antigen stability through epithelial barriers, and the absence of effective adjuvants that help stimulate cell-mediated immunity (16–18). Further, mucosal delivery of vaccine antigens is necessary to induce protective immunity (16, 17).

Nanoparticle-based vaccines are attractive as molecule carriers, as they have been shown to protect the antigen from degradation, facilitating its uptake by antigen-presenting cells (APCs), depot formation, and codelivery of antigens (16–19). In particular, gold nanoparticles (AuNPs) have been utilized to deliver several biomolecules, including codelivery of multiple proteins or DNA antigens, given their intrinsic physicochemical properties and rigid surface (20–25). These particles offer a means to overcome the hurdle of increasing immunogenicity without compromising the safety and tolerability of one or multiple antigens (20, 21, 24, 25). AuNPs have garnered interest in antigen delivery given their high biocompatibility, tunable physicochemical characteristics, such as their size or shape, and the ability to carry multiple antigens on a rigid surface (19, 21, 24–30). Therefore, nanoparticle-based vaccine platforms represent a promising characteristic to surmount some of the challenges of classical vaccine development to elicit strong humoral and cell-mediated immunity (25, 28).

The main objective of the current study was to exploit the use of an AuNP-based platform to deliver an optimal multivalent vaccine formulation while understanding the immunological responses associated with protection against a B. pseudomallei challenge. We synthesized and intranasally delivered a glycoconjugate moiety on AuNPs using several *Burkholderia-*specific antigens along with the LPS from B. thailandensis. Immunization with a combination formulation containing OpcP and OpcP1 (AuNP-Combo2-LPS) afforded complete protection against lethality. Animals immunized with the AuNP-Combo2-LPS had robust antigen-specific lung IgA responses and serum IgG, with elevated IgG_2c_ titers. In addition, sera from AuNP-Combo2-LPS-immunized mice promote antibody-dependent opsonophagocytosis and reduced bacterial survival in primary macrophages. Splenocytes from AuNP-Combo2-LPS-immunized mice showed elevated levels of IFN-γ, tumor necrosis factor alpha (TNF-α), IL-2, IL-17A, and IL-10 upon antigen restimulation. Overall, our study shows that AuNP-coupled glycoconjugate vaccine immunization is associated with antigen-specific humoral and cell-mediated responses associated with protection against B. pseudomallei. Our data highlight the evaluation of two protective antigens, OpcP and OpcP1, against B. pseudomallei and expands on the rationale for the delivery of multiple antigens using an AuNP platform as a means of inducing both humoral and cell-mediated responses.

## RESULTS

### Immunization with AuNP-OpcP-LPS or AuNP-OpcP1-LPS provides protection against an intranasal challenge with B. pseudomallei K96243.

**FIG 1:**
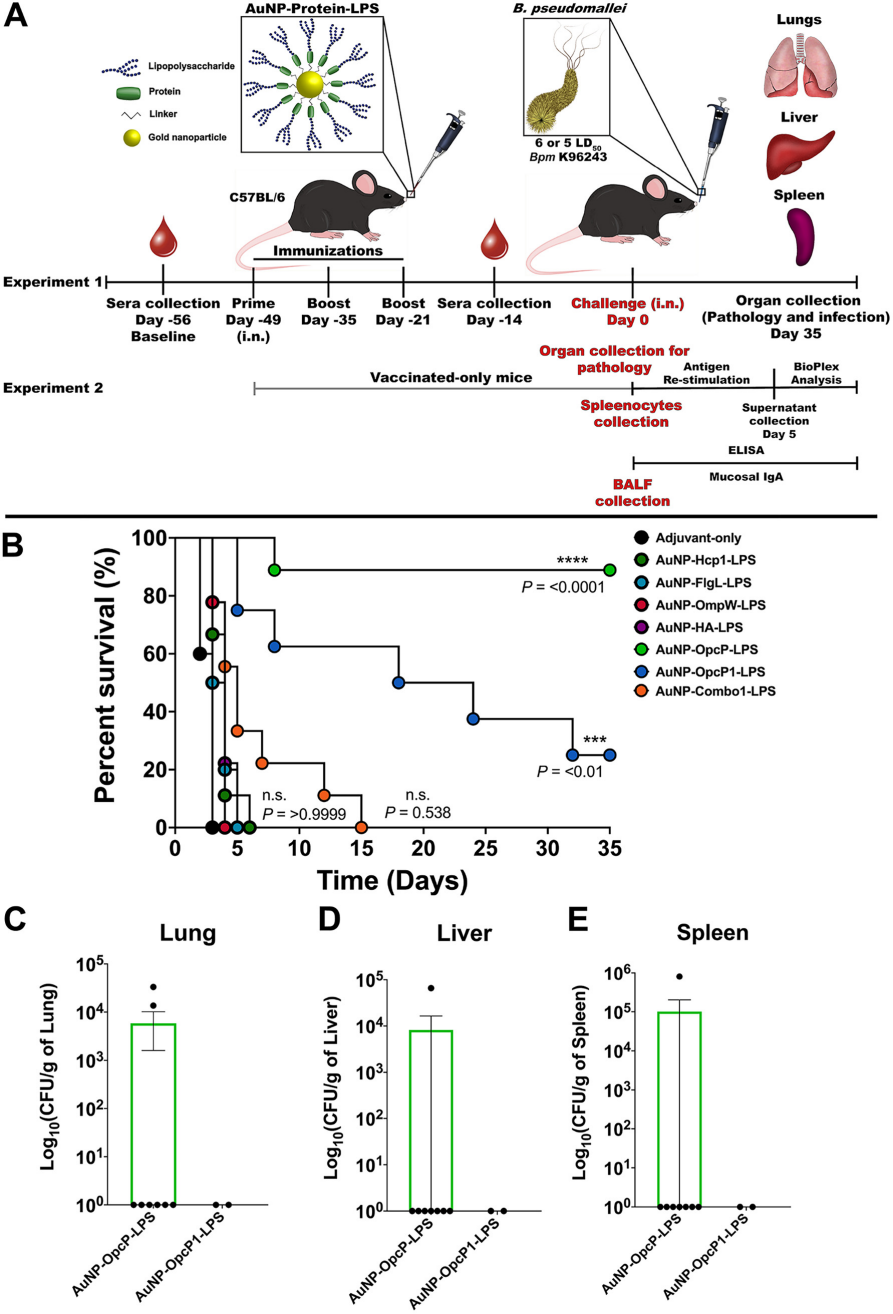
Increased survival from AuNP-OpcP-LPS- or AuNP-OpcP1-LPS-immunized mice against a lethal intranasal challenge with B. pseudomallei K96243. (A) Graphical representation of the vaccination, challenge, and organ/tissue collection timeline. Two separate groups of C57BL/6 mice (at least *n* = 9) were immunized intranasally 3× in 2-week intervals with formulations containing 10 μg of protein, 10 μg of LPS, and 20 μg of CpG ODN 2395. In experiment one, a combination formulation (AuNP-Combo1-LPS) included equivalent amounts of protein (Hcp1, OmpW, OpcP, OpcP1, FlgL, and HA) from each candidate for a total of 10 μg of protein. A second animal experiment (at least *n* = 9) was conducted to evaluate histopathology, cell-mediated response, and BALF from vaccinated-only animals. (B) In experiment one, 3 weeks after the last vaccination, animals were challenged with 6 LD_50_ (9.0 × 10^4^ CFU per mouse) of B. pseudomallei K96243. (C to E) Lungs (C), livers (D), and spleens (E) of surviving animals were collected at 35 days postinfection to evaluate bacterial infection. Bacterial load was determined per gram of tissue, and representative panels for colonization are shown on log scale. All colonization data are shown as means ± standard errors of the means (SEM) of results determined per group. Statistical analyses were determined using the Kaplan-Meier method, followed by log rank test. Levels of significance compared to the adjuvant-only group: ***, *P < *0.001; ****, *P < *0.0001. ns, not significant.

To test the protective properties of several immunogenic proteins when delivered intranasally, we conducted an *in vivo* protection study against an inhalational challenge of B. pseudomallei K96243, using six proteins that we have previously identified (31) (Fig. 1A). We recombinantly expressed and purified each protein by affinity chromatography (Hcp1, OmpW, OpcP, OpcP1, FlgL, and hemagglutinin [HA]) and coupled them to the surface of 15-nm gold nanoparticle (AuNPs) (see Fig. S1 in the supplemental material). In addition, we coupled the lipopolysaccharide (LPS) of B. thailandensis to the protein-decorated AuNPs to incorporate a repeating glycoconjugate moiety onto AuNPs (Fig. S1). We intranasally (i.n.) immunized C57BL/6 mice with three 50-μl doses at 2-week intervals with individual AuNP-protein-LPS candidates containing 10 μg of protein, 10 μg of LPS, and 20 μg of CpG (final concentrations; Fig. 1A). A group of mice also received a combination of equal volumes of individually coupled glycoconjugates (AuNP-Combo1-LPS). Three weeks after receiving the last immunization, animals were challenged with 6 50% lethal dose equivalents (6 LD_50_) of B. pseudomallei K96243. Animals immunized with AuNP-OpcP-LPS or AuNP-OpcP1-LPS showed 90% and 30% protection, respectively, against a lethal inhalational dose of B. pseudomallei K96243 by day 35 postinfection (Fig. 1B). No significant protection was afforded by the AuNP-Combo1-LPS formulation or the conjugates containing candidate Hcp1, FlgL, OmpW, or HA (Fig. 1B). The lung, liver, and spleen of surviving animals were collected at 35 days postinfection, and animals vaccinated with AuNP-OpcP-LPS or AuNP-OpcP1-LPS showed low to no bacterial counts in the three organs (Fig. 1C to E). Two animals had colonization in the lung with 1 × 10^5^ bacteria, but only one showed bacterial infection in the liver and spleen (Fig. 1C to E). These results demonstrate that AuNP-OpcP-LPS and AuNP-OpcP1-LPS are effective antigens to protect against a lethal dose of B. pseudomallei K96243 when delivered intranasally.

10.1128/mBio.01227-21.1 FIG S1 Protein and LPS purification and synthesis of AuNP-linked glycoconjugates validation. (A) Graphical representation of AuNP-linked glycoconjugate, including different components. (B) Coomassie-stained SDS-PAGE gel using 2.5 μg of each protein. (C) Western blot of recombinantly expressed protein antigens after purification, using 0.25 μg of each candidate: Hcp1 (18 kDa), OmpW (29 kDa), OpcP (39 kDa), OpcP1 (41.6 kDa), FlgL (42 kDa), and hemagglutinin (HA) (79.6 kDa). (D) Silver-stained gel of B. thailandensis lipopolysaccharide (2 μg) and Western blot (0.2 μg) using an LPS-specific polyclonal antibody showing the separation of the lipid A and core segments from the O antigen. (E) Transmission electron micrographs of bare spherical 15-nm AuNPs before conjugation and representative particles after conjugation to 5 μg/ml OpcP. The top right inset shows a magnified view of each particle. Scale bar, 20 nm. (F) UV-vis spectroscopy summary of protein and LPS added. Download FIG S1, TIF file, 0.4 MB. Copyright © 2021 Tapia et al. 2021 Tapia et al. https://creativecommons.org/licenses/by/4.0/ This content is distributed under the terms of the Creative Commons Attribution 4.0 International license.

### Immunization with refined AuNP-Combo2-LPS formulation containing OpcP/OpcP1 provides enhanced protection against inhalational B. pseudomallei challenge.

**FIG 2:**
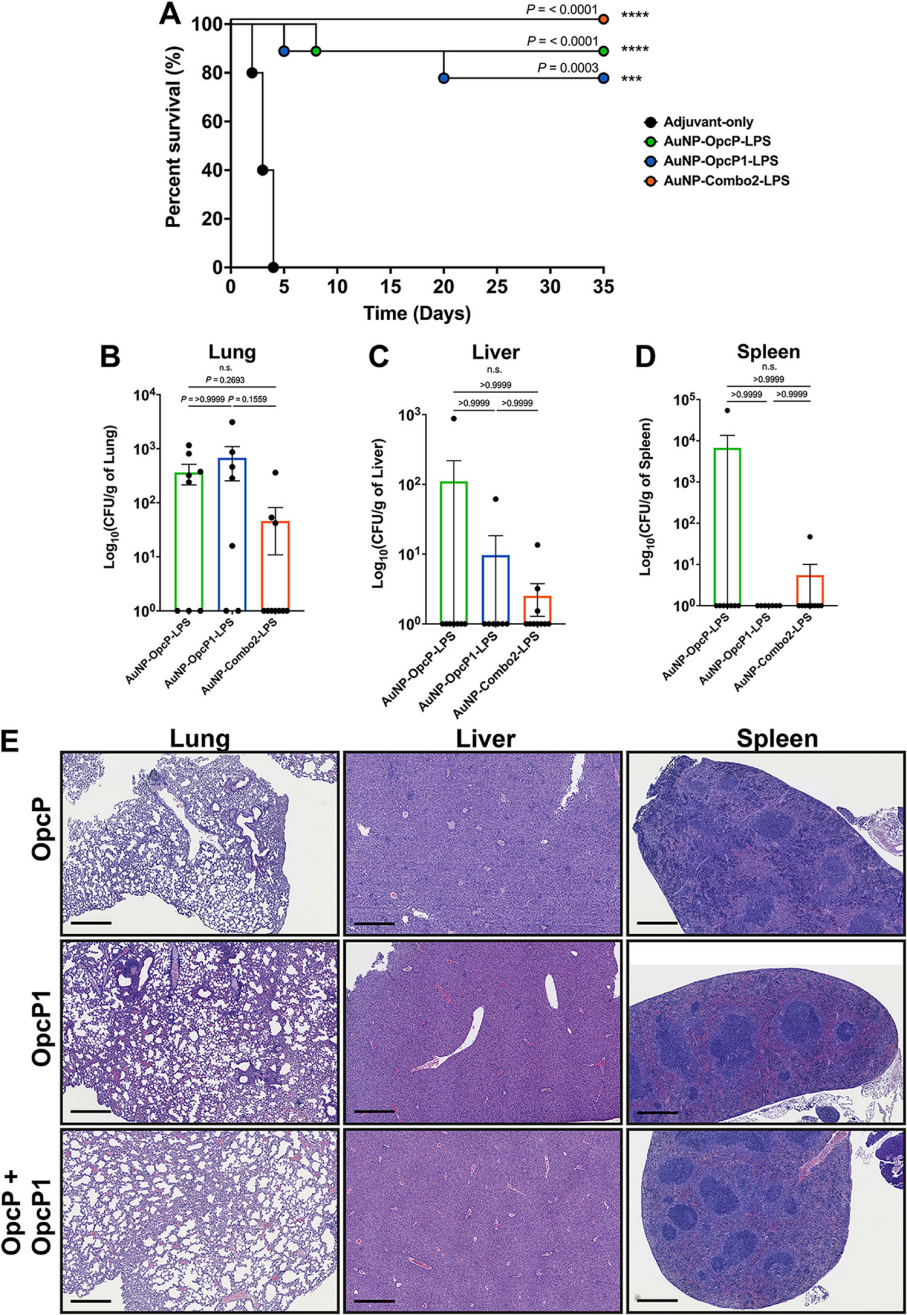
Intranasal immunization with optimized formulation (AuNP-Combo2-LPS) provides enhanced protection against inhalational melioidosis. C57BL/6 mice (at least *n* = 9 ) were immunized as described for Fig. 1. The AuNP-Combo2-LPS vaccinated group contained equivalent amounts of protein (OpcP and Opcp1) from each candidate for a total of 10 μg of protein. (A to D) After intranasal challenge with 5 LD_50_ (7.5 × 10^4^ CFU per mouse) of B. pseudomallei K96243 (A), the lungs (B), livers (C), and spleens (D) of surviving animals were collected at 35 days postinfection, and bacterial enumeration was performed. Bacterial load was determined per gram of tissue, and representative panels for colonization are shown on a log scale. (E) Histopathological analysis from lungs, livers, and spleens of a representative mouse from each surviving group. Lung sections from the AuNP-OpcP-LPS vaccination group showed increased pathological findings compared to the AuNP-OpcP1-LPS and AuNP-Combo2-LPS groups. The liver sections from the AuNP-OpcP-LPS immunization group showed the most inflammation and evidence of liver injury. Spleen sections from surviving animals all showed similar histopathological findings, with the most pronounced in the AuNP-OpcP-LPS vaccine group. At the endpoint of the experiment, tissues were harvested from three mice of the 10 survivors. The tissues were fixed, sectioned, and stained with hematoxylin and eosin. Images are representative of three mice. Scale bar, 500 μm. All colonization data are shown as means ± standard errors of the means (SEM) of results determined per group. Statistical analyses were determined using the Kaplan-Meier method, followed by log rank test. Levels of significance compared to the adjuvant-only group: ***, *P < *0.0005; ****, *P < *0.0001. ns, not significant.

Based on our initial experiment showing a significant increase in protection afforded by the AuNP-OpcP-LPS or AuNP-OpcP1-LPS nanovaccines, we further investigated the protective properties of a combination of these antigens. We performed a similar immunization scheme of three doses in 2-week intervals with subsequent lethal challenge with B. pseudomallei K96243. To evaluate immunization-induced pathology in tissues, the lung, liver, and spleen from three representative animals were evaluated 2 weeks after receiving the last immunization. All tissues from the three different vaccinated groups appeared unremarkable (Fig. S2). Animals immunized with AuNP-OpcP-LPS or AuNP-OpcP1-LPS had a significant increase in survival following a 5 LD_50_ challenge of B. pseudomallei K96243, with 90% (8/9) and 80% (7/9) protection at day 35 postinfection, respectively (Fig. 2A). Further, animals that received a combination formulation with equivalent amounts of AuNP-OpcP-LPS and AuNP-OpcP1-LPS showed complete protection against the lethal dose, with 100% (10/10) of animals surviving to day 35 postinfection (Fig. 2A). Although related in protein predicted function and in molecular weight, we found that OpcP and OpcP1 only share 39% identity and 54% similarity. We collected the lungs, livers, and spleens from surviving animals to assess bacterial infection and histopathology from surviving animals. Mice immunized with AuNP-OpcP-LPS or AuNP-OpcP1-LPS showed some bacterial colonization, with the highest numbers being 1 × 10^3^ CFU/g of tissue in the lungs (Fig. 2B). However, most animals, except for one in each group, did not have any colonization of the liver (Fig. 2B), and only one mouse from the AuNP-OpcP-LPS immunization group showed spleen colonization (Fig. 2C). Animals immunized with the AuNP-Combo2-LPS formulation showed lower lung and liver bacterial counts, and only a single mouse displayed some colonization in the spleen (Fig. 2C and D).

10.1128/mBio.01227-21.2 FIG S2 Histopathology analysis from vaccinated-only mice. Histopathological analysis from lungs, livers, and spleens of a representative mouse immunized intranasally with AuNP-OpcP-LPS (top), AuNP-OpcP1-LPS (middle), or AuNP-Combo-LPS (lower). Two weeks after the last immunization, organs from three representative animals were harvested and fixed in 10% formalin. The tissues were fixed, sectioned, and stained with H&E. All animal tissue sections showed similar findings as reported in the postchallenge groups but to a lesser degree. Images are representative of three mice. Scale bar, 500 μm. Download FIG S2, TIF file, 0.9 MB. Copyright © 2021 Tapia et al. 2021 Tapia et al. https://creativecommons.org/licenses/by/4.0/ This content is distributed under the terms of the Creative Commons Attribution 4.0 International license.

Histopathology analysis of the lung sections from the AuNP-OpcP-LPS vaccination group showed increased interstitial pneumonia and lung consolidation compared to the OpcP1 and combo groups (Fig. 2E, left). In addition, AuNP-OpcP-LPS-immunized mice had more parabronchial and perivascular lymphoid aggregates than the other groups postchallenge (Fig. 2E, left). The liver sections from the AuNP-OpcP-LPS immunization group showed inflammation and evidence of liver injury (Fig. 2E, center). There was mixed portal and lobular inflammation that consisted of both lymphocytes and neutrophils (Fig. 2E, center). Two of three mice showed extramedullary hematopoiesis, which is evidence of liver injury. Livers from these animals also had granulomatous inflammation that was composed of activated histiocytes (Fig. 2E, center). These features were minimal to absent in the OpcP1 and combo postchallenge groups. The sections of spleen from the different immunization groups all showed similar histopathologic changes, although AuNP-OpcP-LPS-immunized mice had increased expansion of the marginal zone and atrophy of the germinal centers compared to the other groups (Fig. 2E, right). Similar histologic findings were observed in all the groups; however, these were at various degrees and most pronounced in the AuNP-OpcP-LPS group (Fig. 2E, right). Together, these results demonstrate that the AuNP-Combo2-LPS vaccine provides complete protection against lethality, with bacterial infection contained in the lung of infected mice.

### Individual AuNP-protein-LPS formulations elicit robust protein- and LPS-specific humoral responses but lower OpcP and OpcP1 antibody titers from the AuNP-Combo1-LPS formulation.

**FIG 3:**
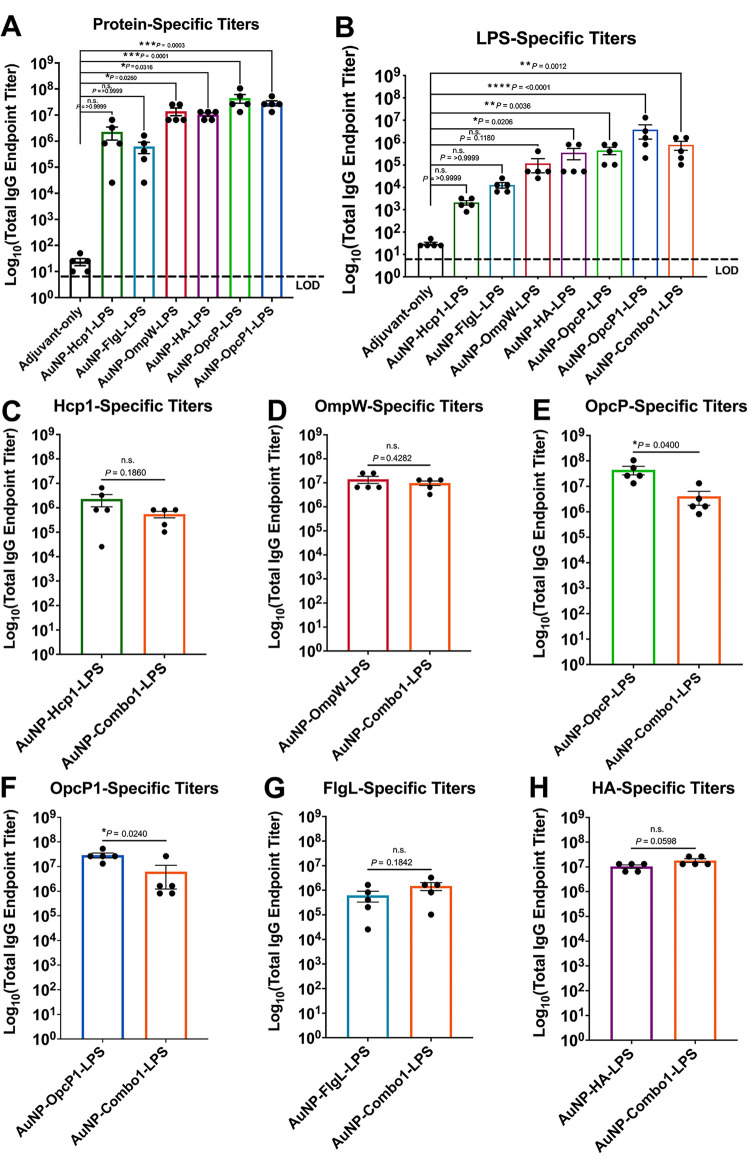
Immunization with individual AuNP-protein-LPS candidates results in robust systemic antigen-specific specific humoral responses but reduced OpcP- and OpcP1-specific antibody response from AuNP-Combo1-LPS vaccination. Total protein and LPS-specific IgG antibody responses. Protein (A)- and LPS (B)-specific total IgG titers were assessed by ELISA, with endpoint titers defined as twice the standard deviation (SD) of the levels measured for naive sera. (C to H) Serum samples taken from the mice immunized with the AuNP-Combo1-LPS formulation were used to assay protein-specific total IgG antibody titers. All antibody data are expressed as means ± SEM of results from at least 5 mice per group and analyzed in triplicate. Significant differences between total IgG protein- or LPS-specific titers were determined using a one-way ANOVA followed by Tukey’s *post hoc* test. Significant differences between individual and Combo1 total IgG titers were determined via Student's *t* test. *, *P < *0.05; **, *P < *0.01; ***, *P < *0.001. ns, not significant.

The protection differences between individual and combination vaccines warranted the analysis of the antigen-specific immune responses occurring between the different formulations. We first analyzed antigen-specific total IgG responses from individual and AuNP-Combo1-LPS formulations against each antigen. Immunization with individual AuNP-protein-LPS candidates showed significantly higher total antigen-specific IgG titers against their corresponding protein or LPS from the AuNP-OpcP-LPS and AuNP-Opcp1-LPS groups compared to adjuvant-only immunized animals (Fig. 3A and B). In addition, AuNP-Combo1-LPS was able to produce elevated LPS-specific total IgG titers (Fig. 3B). To evaluate differences between the individual vaccine formulations and the AuNP-Combo1-LPS group, we measured IgG titers against each protein. Animals immunized with the AuNP-Combo1-LPS formulation showed similar IgG titers against Hcp1 (Fig. 3C), OmpW (Fig. 3D), FlgL (Fig. 3G), and HA (Fig. 3H). However, total IgG titers from the AuNP-Combo1-LPS vaccine showed lower OpcP- and OpcP1-specific titers (Fig. 3E and F). Together, these results confirm that AuNP-protein-LPS vaccination induced robust antigen-specific humoral responses and may explain the reduced protective capacity of the AuNP-Combo1-LPS formulation.

### AuNP-Combo2-LPS formulation induces robust antigen-specific serum antibody response and promotes opsonophagocytosis by primary macrophages.

**FIG 4:**
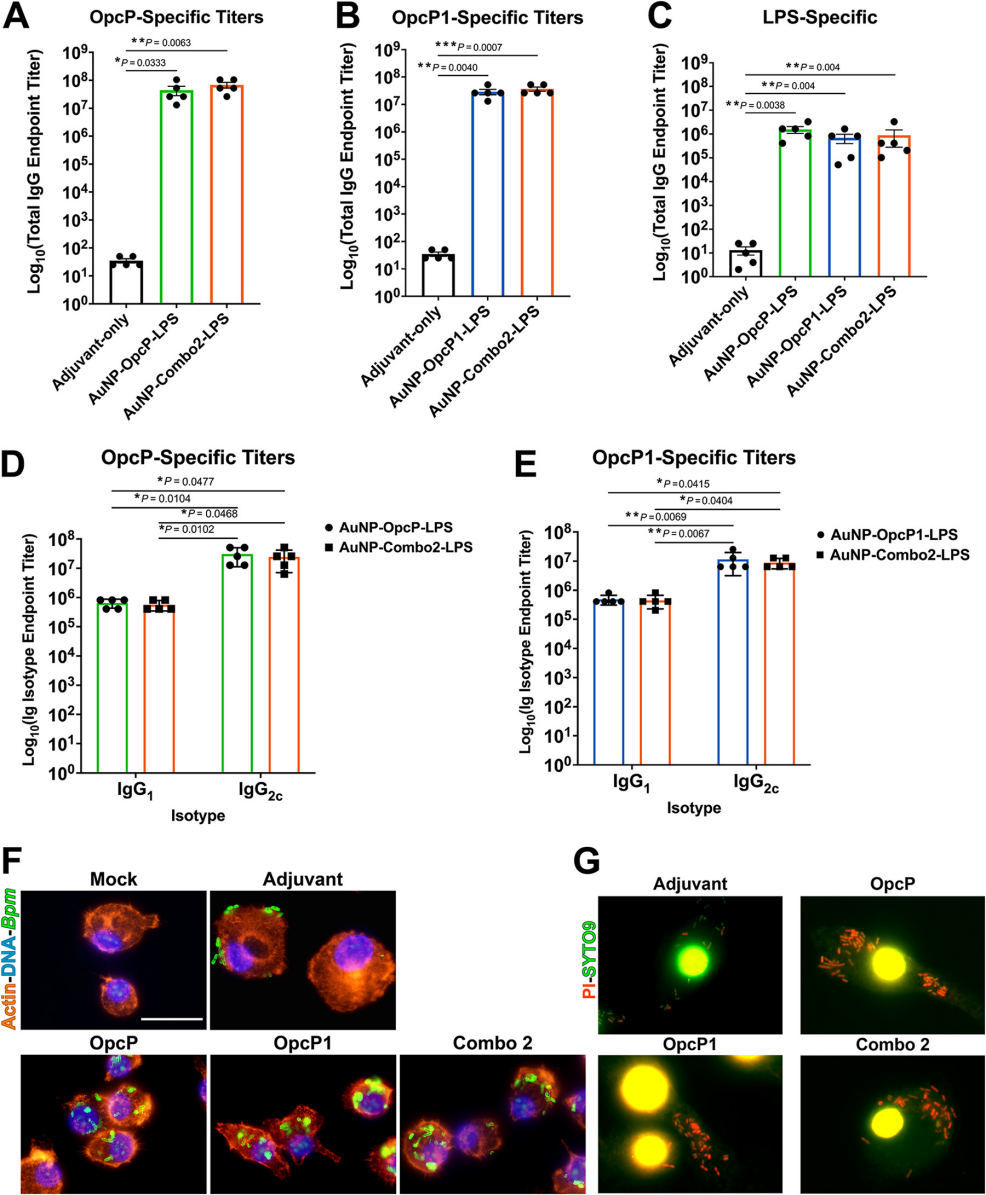
Immunization with optimized AuNP-Combo2-LPS formulation elicits sustained antigen-specific humoral responses associated with increased opsonophagocytosis by macrophages. Protein- and LPS-specific IgG and isotype antibody responses were measured. OpcP (A)-, OpcP1 (B)-, and LPS (C)-specific total IgG titers were assessed by ELISA, with endpoint titers defined as twice the standard deviation (SD) of the levels measured for naive sera. OpcP (D)- and OpcP1 (E)-specific IgG_1_ and IgG_2c_ isotype antibody titers. Serum samples taken from the mice immunized with the AuNP-Combo2-LPS formulation were used to assay protein- and LPS-specific total IgG as well as isotype antibody titers. (F) Fluorescence microscopy analysis of primary murine macrophages 2 h after B. pseudomallei K96243 infection in the presence of immune serum (from OpcP, OpcP1, and Combo2 vaccine groups). After infection, cells were fixed, permeabilized, stained with phalloidin-rhodamine (actin) or DAPI (bacteria and cell nuclei), and examined by immunofluorescence (serum anti-LPS followed by a rabbit anti-mouse Alexa Fluor-488). (G) LIVE/DEAD *Bac*Light-stained primary murine macrophages infected with B. pseudomallei K96243 for 2 h in the presence of sera from each immunization group. Panels below each group represent magnifications (10×) of the images on top. Images were taken using an Olympus BX51 upright fluorescence microscope (60×) and processed using Image J software. Scale bars, 25 μm. All antibody data are expressed as means ± SEM of results from at least 5 mice per group and analyzed in triplicate. Differences in protein-specific total IgG titers or LPS-specific IgG titers between groups were determined using a one-way ANOVA followed by Tukey’s *post hoc* test. *, *P < *0.05; **, *P < *0.01; ***, *P < *0.001.

Given the protection differences afforded by the two different vaccine combinations, we analyzed the antigen-specific total and isotypic antibody differences. We measured total IgG antibody responses elicited by the individual vaccine formulations as well as from AuNP-Combo2-LPS. Animals immunized with the different formulations showed robust total protein-specific IgG responses, with antibody titers of 10^7^ to 10^8^ (Fig. 4A and B). LPS-specific serum IgG titers also showed a robust response in mice immunized with individual formulations, with titers of 10^6^ (Fig. 4C). We showed that immunized animals with the individual and AuNP-Combo2-LPS formulations showed a significantly higher antigen-specific IgG antibody titer than an adjuvant-only control (Fig. 4A to C). Furthermore, protein-specific isotype titers showed higher IgG_2c_ levels than IgG_1_ in animals immunized with either individual formulations or the AuNP-Combo2-LPS (Fig. 4D and E). No significant differences in LPS-specific isotype titers were observed from the different immunization groups (Fig. S3). To test the functionality of these serum antibodies, we analyzed whether the antibodies promote antibody-mediated bacterial opsonophagocytosis. Primary murine macrophages were infected with B. pseudomallei that was previously incubated with immune serum from the vaccination groups. After infection, macrophages were fixed, permeabilized, and analyzed using immunofluorescence microscopy. A higher number of bacteria were internalized by macrophages in the presence of serum from animals immunized with AuNP-OpcP-LPS, AuNP-OpcP1-LPS, or AuNP-Combo2-LPSthan bacteria incubated with naive serum (Fig. 4F). Using bacterial live/dead staining, we evaluated internalization of the bacteria by macrophages. After 2 h of infection, most of the internalized bacteria, in the presence of immune sera, were nonviable compared to naive sera and were visualized by the incorporation of propidium iodide (Fig. 4G). These data demonstrate that the AuNP-Combo2-LPS formulation induced a strong antigen-specific antibody response, promoting opsonophagocytic activity and inducing higher IgG_2c_ titers.

10.1128/mBio.01227-21.3 FIG S3 LPS-specific isotype titers in serum of AuNP-linked glycoconjugate immunized mice. Sera from immunized animals were collected 2 weeks after the last immunization. LPS-specific IgG_1_ and IgG_2c_ antibody isotype titers were assessed by ELISA, with endpoint titers defined as twice the standard deviation (SD) of the levels measured for naive sera (adjuvant only). All antibody data are expressed as mean ± SEM of results from at least 5 mice per group and analyzed in triplicate. Significant differences between LPS-specific isotype titers were determined using a two-way ANOVA followed by Tukey’s *post hoc* test. ns, not significant. Download FIG S3, TIF file, 1.2 MB. Copyright © 2021 Tapia et al. 2021 Tapia et al. https://creativecommons.org/licenses/by/4.0/ This content is distributed under the terms of the Creative Commons Attribution 4.0 International license.

### Intranasal immunization with AuNP-Combo2-LPS induces robust antigen-specific lung IgG and IgA responses.

**FIG 5:**
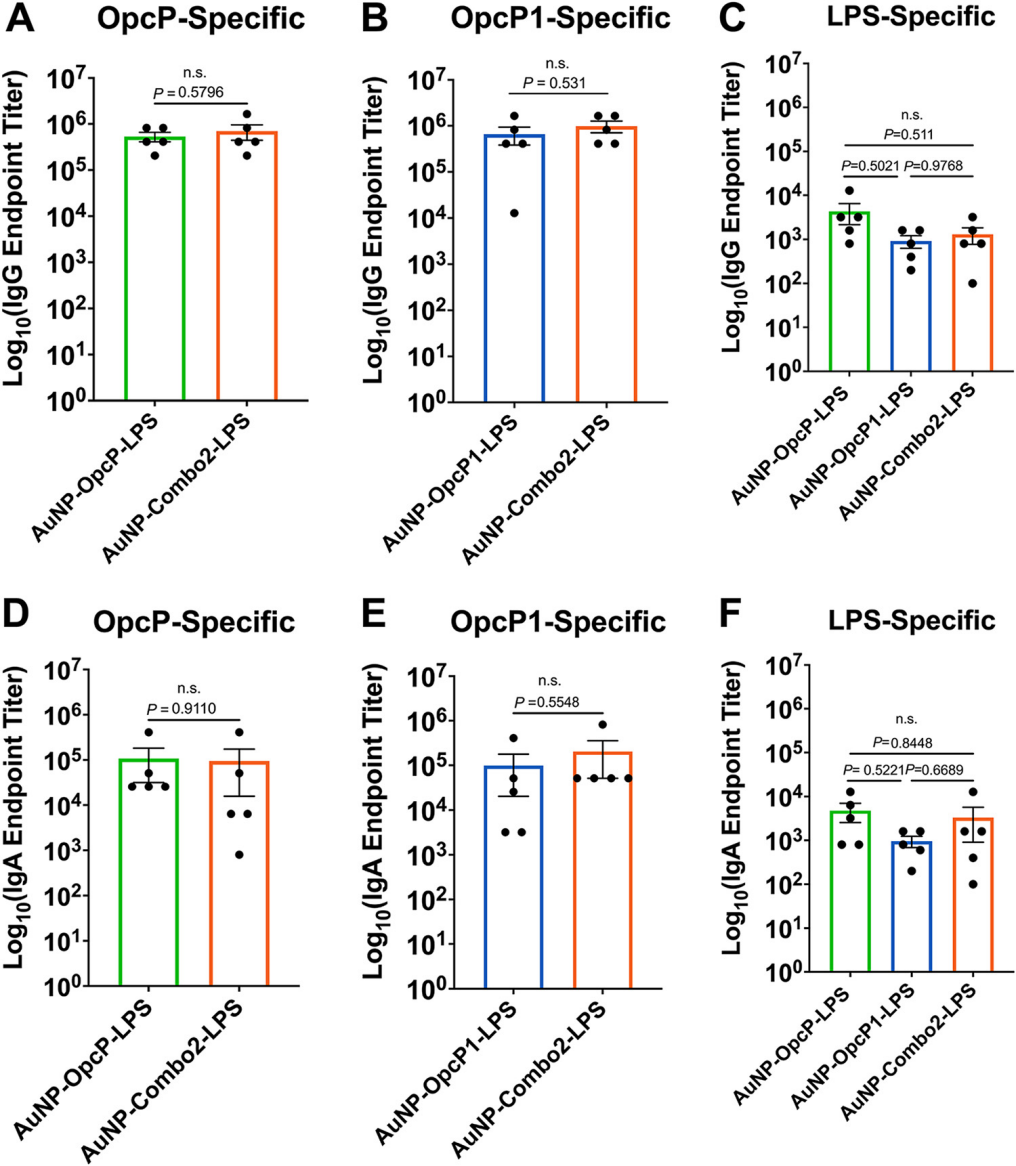
Immunization with optimized AuNP-Combo2-LPS formulation elicits robust lung total IgG and IgA responses. Total IgG and IgA protein-specific antibody responses were analyzed from BALF of immunized animals 3 weeks after the last immunization. (A to C) OpcP-, OpcP1-, and LPS-specific total IgG titers were assessed by ELISA, with endpoint titers defined as twice the standard deviation (SD) of the levels measured from adjuvant-only immunized animals. (D to F) OpcP-, OpcP1-, and LPS-specific IgA serum responses were analyzed by ELISA. Serum samples taken from the mice immunized with the AuNP-Combo2-LPS formulation were used to assay protein-specific total IgG and IgA antibody titers. All antibody data are expressed as means ± SEM of results from at least 5 mice per group and analyzed in triplicate. Significant differences between total IgG and IgA protein for protein-specific titers were determined by a Student's *t* test, and LPS-specific titers were determined using a one-way ANOVA followed by Tukey’s *post hoc* test. ns, not significant.

We measured IgG and IgA antibody responses in the bronchoalveolar lavage fluid (BALF) of immunized mice to understand tissue-specific protective responses. Antigen-specific lung total IgG (Fig. 5A to C) and IgA titers (Fig. 5D to F) were evaluated 3 weeks after the final intranasal immunization. Animals receiving individual formulations (AuNP-OpcP-LPS or AuNP-OpcP1-LPS) showed strong antigen-specific total IgG responses in the lung (Fig. 5A to C), with levels around 10^6^ for protein-specific and 10^3^ for LPS-specific responses (Fig. 5A to C). Similarly, animals immunized with single formulations showed robust antigen-specific lung IgA responses with titers of 10^5^ and 10^4^ against protein or LPS, respectively, compared to control mice (Fig. 5D to F). These antigen-specific IgG and IgA responses were equivalent to those seen in animals immunized with AuNP-Combo2-LPS (Fig. 5A to F). These results further validate that the gold nanovaccine induces strong humoral responses in the lung after intranasal immunization.

### Gold-linked glycoconjugate (AuNP-Combo2-LPS) vaccine induces an antigen-specific mixed T_H_1-T_H_17-biased cytokine response to protein restimulation.

**FIG 6:**
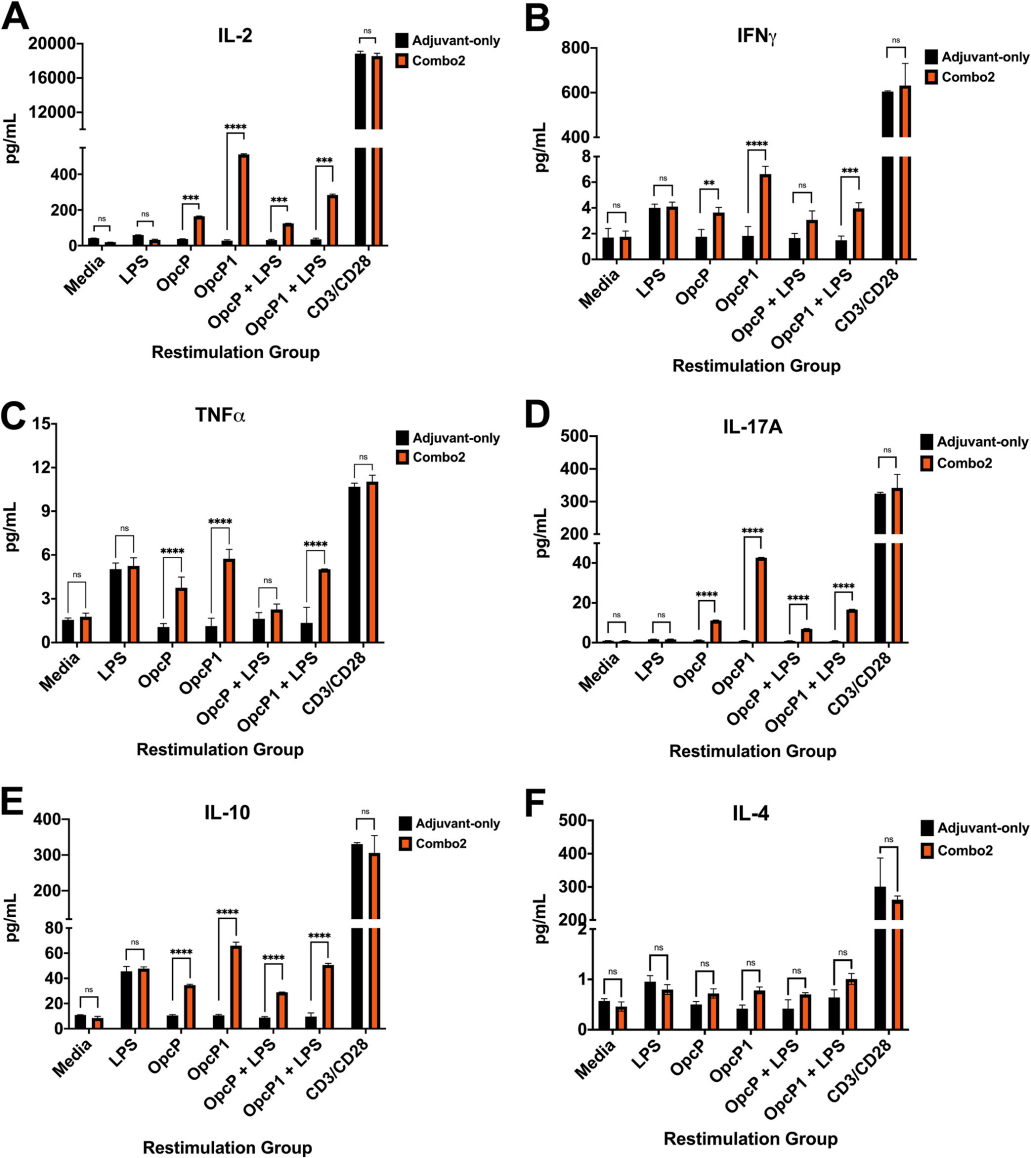
Mice immunized with AuNP-Combo2-LPS produce a robust antigen-specific T_H_1-T_H_17 cytokine profile correlated with antigen-specific responses. Antigen-specific cytokine profile production from AuNP-Combo2-LPS splenocyte upon stimulation 3 weeks postimmunization was studied. Supernatants were analyzed for production of IL-2 (A), IFN-γ (B), TNF-α (C), IL-17A (D), IL-10 (E), and IL-4 (F). Mice (five per group) were immunized with 10 μg of protein and 10 μg LPS adjuvanted with 20 μg of CpG. Two boosts were given 2 weeks apart on days −35 and −21, and spleens were harvested 3 weeks after the final boost. Spleen cells from AuNP-Combo2-LPS-immunized mice were stimulated for 5 days with protein, LPS, or both. Splenocyte-stimulated media or anti-CD3/CD28 antibodies served as controls. Supernatants were assayed for cytokine expression using a murine BioPlex ELISA kit. All cytokine data are expressed as means ± SEM of results from at least 5 mice per group and analyzed in triplicate. Significant differences were determined using a two-way ANOVA followed by Tukey’s *post hoc* test. *, *P < *0.05; **, *P < *0.01; ***, *P < *0.001, ****, *P < *0.0001. ns, not significant.

Given our previous observations showing induction of a T_H_1-biased response with antibody IgG_2c_ subtype after AuNP-Combo2-LPS immunization, we determined if our vaccine activates antigen-specific T-cell responses. Spleens from AuNP-Combo2-LPS or adjuvant-only immunized mice (*n* = 5) were collected on day 21 postimmunization, and single-cell suspensions were cultured in the presence of different antigens. Splenocytes from AuNP-Combo2-LPS or adjuvant-only immunized animals were stimulated with OpcP, OpcP1, B. thailandensis LPS, a combination of OpcP and LPS or OpcP1 and LPS, αCD3/αCD28 antibody-conjugated beads, or mock solution (medium control). Five days after restimulation, cell supernatants were collected and used to measure the production of cytokines. Our results show a significant increase in IL-2, IFN-γ, TNF-α, IL-17A, and IL-10 cytokine production in response to OpcP or OpcP1 restimulation (Fig. 6A to E). In contrast, no significantly higher differences were seen after the addition of LPS to either OpcP or OpcP1 (Fig. 6A to E). Furthermore, no protein- or LPS-specific significant differences in IL-4 production from splenocytes upon restimulation were measured (Fig. 6F). These data further demonstrate induction of a mixed protein-specific T_H_1-T_H_17-biased response after intranasal vaccination with AuNP-Combo2-LPS.

## DISCUSSION

Current evidence indicates that protection against melioidosis, both in humans and animal models, requires robust humoral and cellular adaptive immunity (5–8, 10, 13–15, 32–37). However, efficient vaccine delivery platforms capable of eliciting robust, long-lasting, and specific immune responses while maintaining a safe profile are limited (17, 18, 24, 27). The development of potent and safe vaccine platforms that help promote cellular immunity against intracellular bacteria remains a challenge and warrants further investigation. Nanoparticle-based vaccines, mainly those utilizing gold nanoparticles, offer a means of delivering multiple antigens on the same surface with greater efficacy to lymphoid organs than soluble antigens (17, 18, 24, 26, 28, 32). Although the mechanisms promoting increased immune responses are not fully understood, they involve enhancing antigen stability to cross mucosal barriers and increase antigen processing and presentation upon antigen-presenting cell (APC) internalization (17, 18, 24, 26, 28, 32). The stable conjugation of a protein-LPS moiety onto the surface of AuNPs seeks to preserve important antigenic molecular structures critical for eliciting robust humoral and cell-mediated immunity in a multivalent display of antigens on a rigid surface. The design of an AuNP-delivered glycoconjugate vaccine seeks to elicit robust immune responses against multiple antigens to increase the protective efficacy and security of this vaccine platform (26, 30, 32). To exploit this idea, we covalently conjugated a protein-LPS glycoconjugate motif on the surface of 15-nm AuNPs via a small hydrophobic linker (16-MHDA). The lipopolysaccharide (LPS) of B. thailandensis, composed of the O-antigen molecule (OAg) and the lipid A moiety, were coupled to AuNP-protein conjugates to broaden the protective response (34, 35, 38). The LPS of B. thailandensis, a T-cell-independent antigen, is expected to engage T-cell help, allowing for B-cell memory development and the production of antibody responses (39). The lipid A region of the B. thailandensis LPS structure could act as an adjuvant by stimulating the production of inflammatory cytokines (Fig. 6) (35, 38, 40). Furthermore, the final vaccine formulation contained the toll-like receptor 9 (TLR9) agonist, CpG ODN 2395, to augment and divert the immunogenicity of the protein or LPS antigens to a T_H_1-biased response (17).

Given that inhalational exposure to B. pseudomallei presents the most lethal route of infection, a vaccine that elicits robust mucosal responses is needed to fully protect against inhalational challenge and represents the most appealing strategy for inducing specific immune responses in mucosal tissues (5). However, vaccines that can be delivered in the mucosa presented challenges of antigen uptake, rapid clearance, size restriction for amenability to cross the epithelium, few mucosal adjuvants available, etc. (16). Therefore, AuNPs ameliorate some of the mucosal subunit vaccines' challenges by affording a safe platform for antigen stability, uptake, and delivery. We synthesized individual gold-linked glycoconjugates using six *Burkholderia*-specific protein antigens that were derived from our previous predictions using bio- and immunoinformatic reverse vaccinology analysis (31). We have previously shown that several of these candidates provided increased protection in a murine model of glanders (B. mallei infection) and promoted different protection levels when delivered using a similar immunization route (31, 41). However, we wanted to evaluate their protective efficacy against B. pseudomallei, develop a refined formulation, and analyze which humoral and cellular immune responses are elicited. We found that two of these antigens, namely, OpcP and OpcP1, provided a significant increase in protection against B. pseudomallei, but a combination of these in equal ratios (AuNP-Combo2-LPS) afforded 100% mouse survival against inhalational melioidosis. Most of the surviving animals had bacterial infection contained in the lung and few pathological lesions observed compared to the individual AuNP-protein-LPS formulations. The absence of sterilizing immunity could be due to the complex lifestyle of B. pseudomallei, which requires numerous and redundant virulence mechanisms, or the need to broaden the protective response using other conserved and immunogenic antigens. While OpcP (BPSS0879) and OpcP1 (BPSS0708) only share 39% amino acid sequence identity, both proteins are predicted to form porins in the bacterial outer membrane (OM). Interestingly, OpcP is an abundant porin in the OM of B. pseudomallei, comprising up to 11% of its total OM proteins (42). Future studies should aim at defining the function of this protein and the implications in antigen processing. Nonetheless, our data provided the rationale for analyzing the antigen-specific protective marker differences between OpcP and OpcP1.

Our results showed that AuNP-OpcP-LPS and AuNP-OpcP1-LPS were associated with the highest percent survival and maintained strong total IgG responses against protein and LPS antigen alone. However, we observed reduced antigen-specific IgG titers when given in a formulation containing six antigens (AuNP-Combo1-LPS). A reduced immune response against our leading candidates, or a difference in formulation dose from each individual candidate, could provide a plausible explanation for the inability of the AuNP-Combo1-LPS formulation to provide full protection against a B. pseudomallei challenge. Our refined vaccine AuNP-Combo2-LPS showed that antigen-specific serum IgG titers were equivalent against both protein and LPS compared to the individual vaccines. Our data also demonstrate the ability of glycoconjugate-coupled AuNPs to elicit strong humoral responses against multiple antigens, including protein and LPS. This observation is important, as melioidosis convalescent patients have higher OAg-specific antibody titers correlated with a higher degree of survival (9, 32, 33). Our data also demonstrate the ability of AuNP-Combo2-LPS intranasal vaccination to elicit systemic IgG and lung IgA responses, correlating with protection. This is critical, as melioidosis survivors have higher LPS-specific IgA titers than nonsurvivors (32). Our results further strengthen the hypothesis that antibodies targeting the LPS of B. thailandensis are associated with protection against B. pseudomallei infection. Future studies should focus on determining the immunological function of LPS in the context of vaccination to determine if this molecule can act as an adjuvant or an antigen. However, although LPS-specific titers alone may not correlate to provide protection, as groups that did not induce protection still had significantly higher antibody LPS titers, protein-specific titers may be critical for full protection. These results further highlight that gold-linked glycoconjugate intranasal vaccine immunization induces strong systemic and mucosal humoral responses. This also suggests that humoral protective immunity could protect against bacteria at different stages of infection, including inhibition of bacterial adherence onto the mucosal epithelium, preventing subsequent dissemination. Bacterial opsonophagocytosis in the presence or absence of immune sera showed an increase in bacterial uptake when serum from AuNP-OpcP-LPS or AuNP-OpcP1-LPS was added, a function that was also observed with the AuNP-Combo2-LPS formulation. The serum effect was confirmed by the reduced bacterial viability within macrophages. It has been previously shown that sera from convalescent melioidosis patients promote antibody-dependent bacterial uptake and bacterial clearance (33). Our observations strengthen the hypothesis that humoral responses are an essential correlate of protection in melioidosis patients.

To analyze isotypic antibody differences as a measure of immune-biased responses, we determined IgG_2c_ and IgG_1_ isotype titers from individual and AuNP-Combo2-LPS formulations. Higher IgG_2c_ antibody titers in C57BL/6 mice are associated with the induction of a T_H_1-biased response, given that this mouse genetic background does not express the IgG_2a_ isoform (43, 44). Our isotype differences, with higher protein-specific IgG_2c_ titers, suggest a T_H_1-biased immune response, as has been reported previously (44). It has been shown that cell-mediated immunity is important for protecting many intracellular pathogens, including B. pseudomallei (1). The correlation of cell-mediated immunity, especially the production of IFN-γ, with human melioidosis survival prompted the analysis of its induction by our gold-linked glycoconjugate vaccines. Our results from splenocytes of immunized animals exogenously restimulated with antigen demonstrate that protein-specific T-cell responses may contribute to the protection afforded by these vaccines. In response to protein restimulation, splenocytes from AuNP-Combo2-LPS produce higher levels of IFN-γ, IL-2, TNF-α, IL-17A, and IL-10. Interestingly, human peripheral blood mononuclear cells from diabetic melioidosis patients showed reduced IL-17 production upon restimulation, suggesting that IL-17 is playing a significant role in response to B. pseudomallei infection (13). Furthermore, the induction of a mixed T_H_1-T_H_17 response has previously been observed in other *Burkholderia* vaccines, including our own live-attenuated B. mallei vaccine CLH001 (Δ*tonB* Δ*hcp1*) (45, 46). The importance of IFN-γ and IL-17 has broad implications in vaccine design, as they are important in promoting optimal macrophage activation for intracellular pathogen killing and antibody production, respectively (47–49). Future studies should focus in determining the role of effector T_H_17 cells in protection against B. pseudomallei infection. This response could provide an important correlation between gold-linked glycoconjugate vaccines and the ability to elicit robust humoral and cell-mediated immunity (25). Future studies should determine the tissue-specific T-cell-mediated responses and epitope mapping of both OpcP/OpcP1 antigens. In summary, we generated an optimized vaccine strategy against inhalational melioidosis. The resulting optimized combination vaccine (AuNP-Combo2-LPS) displayed full protection against a lethal B. pseudomallei K96243 challenge with unremarkable tissue pathology. We showed that delivery of individual or a combination of gold-coupled glycoconjugates induced robust antigen-specific humoral responses both systemically and at mucosal sites. Finally, we showed that splenocytes from immunized mice produced protein-specific mixed T_H_1-T_H_17 cells upon antigen restimulation. Our study provides a vaccine strategy against B. pseudomallei and an immune-stimulatory platform to induce strong humoral and T-cell-mediated immunity.

## MATERIALS AND METHODS

### Bacterial strains and growth conditions.

Burkholderia thailandensis E264 and B. pseudomallei K96243 were routinely grown aerobically (200 rpm) at 37°C in Luria-Bertani (LB) medium (1% tryptone, 0.5% yeast extract, 0.5% NaCl) and 4% glycerol (LBG), a common medium used for culturing B. pseudomallei. All bacterial cultures were incubated at 37°C for 16 to 18 h. All chemical reagents, unless otherwise noted, were purchased from Sigma-Aldrich.

### Protein purification.

B. mallei-specific proteins Hcp1 (BMAA0742), OmpW (BMA2010), OpcP (BMAA1353), OpcP1 (BMAA1122), FlgL (BMA3336), and hemagglutinin (BMAA1324) were cloned into pET30a(+) protein expression vectors and transformed into BL21(DE3) competent Escherichia coli cells (31). To induce protein expression, overnight cultures were diluted 1:20 in 1 liter of LB broth containing 50 μg/ml kanamycin, grown to an optical density at 600 nm (OD_600_) of 0.6 to 0.8, and induced with 1 mM (final concentration) isopropyl-d-1-thiogalactopyranoside (IPTG). Cultures were centrifuged (4,000 × *g* for 15 min) at 3 h postinduction, and each resulting bacterial pellet was frozen at −20°C. The bacterial pellets were then resuspended in 40 ml of 1× Dulbecco’s phosphate-buffered saline (DPBS) containing 10% glycerol and 25 mM sucrose with a 1 mg/ml final concentration of lysozyme, 0.2% sodium deoxycholate, and a tablet of cOmplete EDTA-free protease inhibitor cocktail (Roche, Germany). This lysate was then sonicated and centrifuged, and the pellet was used for subsequent washes to maximize soluble protein extraction. After centrifugation (20,000 × *g* for 40 min), the supernatant was filter sterilized (0.2-μm pore size; Millipore). Soluble protein extracts were then bound to Talon cobalt (Co^2+^) columns (GE Healthcare, USA) and washed with PBS buffer–50 mM imidazole. Proteins were eluted from affinity columns by applying a 1× PBS buffer with 10% glycerol, 25 mM sucrose, and 250 mM imidazole. Fractions containing soluble protein were collected and pooled before dialysis into PBS containing 10% glycerol and 25 mM sucrose overnight at 4°C. Endotoxin levels were tested using a Pierce LAL chromogenic endotoxin quantification kit (ThermoFisher Scientific, USA) by following the instructions of the manufacturer. The limit of detection for endotoxin is approximately 0.1 EU/ml solution. The purified proteins and protein standards were subjected to a colorimetric bicinchoninic acid (BCA) assay in accordance with the manufacturer’s protocol and were then stored at −80°C until use (Pierce protein assay kit; ThermoFisher Scientific, USA). For protein visualization, 0.25 μg of each protein was run on SDS-PAGE gel by electrophoresis. Gels were transferred to a nitrocellulose membrane for Western blot analysis. A mouse antihistidine antibody (ThermoFisher Scientific, USA) was used (1:5,000), and the reaction mixture was incubated overnight at 4°C; horseradish peroxidase (HRP)-conjugated rabbit anti-mouse IgG was used (1:10,000) as a secondary antibody. Protein bands were visualized by adding ECL substrate (ThermoFisher Scientific, USA), and the results were imaged on film.

### Lipopolysaccharide extraction.

The LPS from B. thailandensis E264 was isolated by the hot phenol extraction method (41). Briefly, a pellet of 4 liters of LB-grown B. thailandensis at stationary phase (24 h at 37°C and 200 rpm) was collected (6,000 × *g* for 15 min) and lysed in the presence of a mixture of 1:1 phenol in water (ThermoFisher Scientific, USA). After lysis at 80°C, phenol was removed by dialysis 4× into ultrapure water and centrifuged (15 min at 6,000 × *g*) to clarify the solution, and the supernatant was lyophilized. The lyophilized solution was resuspended in an aqueous solution containing 10 mM Tris-HCl (pH 7.5), 1 mM MgCl_2_, 1 mM CaCl_2_ and digested with RNase, DNase I, and proteinase K (50 μg/ml each). After clarification (100,000 × *g* for 3 h), the resulting supernatant containing LPS was lyophilized. The sample containing LPS was washed 5× times with 90% ethanol and lyophilized. After lyophilization, the pellet was weighed, resuspended in PBS, and stored at −80°C until use. The purity of LPS was assessed by SDS-PAGE, followed by silver staining by following the manufacturer’s protocol (Pierce color silver stain kit).

### Gold nanoparticle synthesis and coupling.

Spherical 15-nm gold nanoparticles (AuNPs) were synthesized by the Turkevich method (50). Briefly, 1 mM gold(III) chloride trihydrate underwent a reduction reaction with 90 mM sodium citrate dihydrate. Particle size and shape were analyzed by transmission electron microscopy (TEM). To stabilize the conjugation of soluble antigens onto the AuNP surface, 0.1 mM 16-mercaptohexadecanoic acid (16-MHDA) and 0.1% Triton X-100 were added to AuNPs. After 2 h of incubation, this solution was filtered by centrifugation (Amicon Ultra-15; 30-kDa molecular weight cutoff [MWCO]; EMB Millipore), and the procedure was repeated for 2 h to ensure complete coverage. Covalent protein conjugation by carbodiimide synthesis was achieved by adding 20 μg of protein per ml of nanoparticles in the presence of 1 mM DMTMM [4-(4,6-dimethoxy-1,3,5-triazin-2-yl)-4-methyl-morpholinium chloride], as previously described (31). The AuNP-protein conjugation reactions were carried out in 100 mM borate buffer for 12 h. Attachment of 16-MHDA and protein was confirmed by measuring plasmon resonance via UV-visible (UV-Vis) spectroscopy, TEM, and SDS-PAGE, as previously described (31). To conjugate LPS onto the AuNP-protein conjugates, we employed the thiol-maleimide synthesis mechanism. To achieve this, LPS was activated by the addition of 80 mM EMCH [N-ε-maleimidocaproic acid hydrazide] cross-linker in the presence of 40 mM EDC [*N*-(3-dimethylaminopropyl)-*N*′-ethylcarbodiimide hydrochloride] and 10 mM NHS (*N*-hydroxysuccinimide) in 50 mM morpholineethanesulfonic acid buffer (pH 7.0). After 1 h at room temperature, LPS was concentrated to the desired concentration using an Amicon Ultra-15, 30-kDa MWCO. After desalting the LPS in 5 mM EDTA, 20 μg of activated LPS was added per ml of protein-coupled AuNPs previously activated in the presence of 250 μM SATA (S-acetylthioglycolic acid N-hydroxysuccinimide ester) for 1 h at room temperature. After 4 h of incubation, the reaction was quenched with 5 mM N-ethylmaleimide. The AuNP-protein-LPS conjugates were washed 2× with PBS containing 10% glycerol and 25 mM sucrose and concentrated to the desired volume using an Amicon stirred cell containing a 100-kDa-MWCO filter.

### Animal studies.

Female 6- to 8-week-old C57BL/6 mice were purchased from Jackson Laboratories (Bar Harbor, ME, USA) and maintained in an animal biosafety level 3 (ABSL3) facility. Animals were housed in microisolator cages under pathogen-free conditions with food and water available *ad libitum* and maintained on a 12-h light cycle. All animal protocols were reviewed and approved by the Institutional Animal Care and Use Committee (IACUC) of the University of Texas Medical Branch (protocol no. 0503014D). To allow adequate acclimation, mice were housed within the animal facility for 1 week prior to experimentation.

### Immunization and challenge studies.

C57BL/6 mice (at least *n* = 9 per group) were inoculated intranasally (i.n.) three times in 2-week intervals with 50-μl formulations. Animals received each of the AuNP-protein-LPS conjugates. Two combination AuNP formulations were synthesized by mixing equal ratios of individually coupled AuNP-protein-LPS conjugate, AuNP-Combo1-LPS (containing Hcp1, OmpW, OpcP, OpcP1, FlgL, and HA) or AuNP-Combo2-LPS (containing OpcP and OpcP1). Each vaccine formulation contained a total of 10 μg of protein and 10 μg LPS, along with 20 μg of CpG ODN 2395 (InvivoGen, USA). Control groups received 20 μg of adjuvant alone. To evaluate antibody titers, blood was drawn retro-orbitally 2-weeks following the last boost (*n* = 5). To isolate sera, blood was incubated for 30 min at room temperature (RT) to allow clotting and centrifuged (10,000 × *g* for 10 min). Serum was removed and stored at −80°C until use. For assays requiring serum, the sera from immunized animals (*n* = 5) were pooled and stored. Three weeks after administering the last immunization, animals were challenged with a low- or high-dose challenge of B. pseudomallei K96243 in 50-μl samples. The low-dose challenge animals received a dose of 5 LD_50_ (∼7.5 × 10^4^ CFU per mouse), while the high-dose challenge received 6 LD_50_ (9 × 10^4^ CFU per mouse). To enumerate bacterial colonization, the lung/spleen (low-dose challenge) or lung, liver, and spleen (high-dose challenge) were collected. Organs were homogenized in 1 ml of 1× PBS, serially diluted, and plated on LBG agar to quantify bacterial colonization at 37°C for 48 h. The bacterial limit of detection (LOD) was determined to be 1 CFU/organ.

### Histopathology evaluation.

At 15 days postvaccination or 35 days postchallenge, three representative animals from each group were euthanized and their lungs, livers, and spleens were fixed with 10% normal buffered formalin. For histopathological analysis, fixed tissues were embedded in paraffin and sectioned prior to staining with hematoxylin and eosin (H&E). Representative images from each organ from one mouse per group were taken and analyzed. Pathology scoring was performed blindly by a certified pathologist from UTMB, not directly associated with the design of any experiment.

### Detection of antigen-specific antibodies.

Baseline and postvaccinated sera were collected from animals administered adjuvant only, individual AuNP-protein-LPS conjugates, and AuNP-Combo2-LPS formulation 2 weeks after the second boost. Whole blood was collected via retro-orbital bleeding and stored in Microvette tubes without anticoagulant. The sera were separated by centrifugation and stored at −80°C. The protein-specific total IgG, IgG_1_, and IgG_2c_ titers were determined by indirect enzyme-linked immunosorbent assay (ELISA). Bronchoalveolar lavage fluid (BALF) from AuNP-protein-LPS or AuNP-Combo2-LPS immunized mice were collected 3 weeks after receiving the last immunization and stored at −80°C (day 0). Briefly, a microplate (Costar, Cambridge, MA) was coated with each protein or LPS antigen (1 μg/well) in a mixture with 1× PBS (Corning, USA) and maintained at 4°C overnight. Wells were washed twice with washing buffer (0.05% Tween 20–DPBS) and then treated with blocking buffer (0.05% Tween 20, 2% bovine serum albumin [BSA], 1× DPBS) at RT for 2 h. The blocked wells were washed twice before the addition of sample diluent (1% BSA–0.05% Tween 20–1× DPBS). Baseline sera, adjuvant-only BALF, sera from immunized animals, or BALF samples were added to each top dilution well in triplicate, and 2-fold dilutions were performed following incubation at RT for 2 h. Diluted goat anti-mouse IgG, IgG_1_, IgG_2c_, or IgA (Southern Biotech, USA) (1:5,000) was added into each well and then incubated for 3 h after washing. Plates were washed four times prior to addition of tetramethylbenzidine (TMB) substrate solution (Invitrogen, USA). Stop solution (2N H_2_SO_4_) was added, and the samples were immediately read at 450 and 570 nm using a microplate reader (BioTek, USA). The results were reported as the reciprocal of the highest titer, giving an optical density (OD) reading of at least the mean ± 2 standard deviations compared to the baseline sera or adjuvant-only BALF (for lung IgA or IgG). All assays were performed in triplicate, and results are reported as mean reciprocal endpoint titers.

### Macrophage survival assay and fluorescence microscopy.

C57BL/6 murine bone marrow-derived primary macrophages (BMDM) (no. C57-6030; Cell Biologics Inc., Chicago, IL) were routinely grown in complete primary cell culture medium by following the manufacturer’s instructions (no. M3368; Cell Biologics). Cells were incubated at 37°C and 5% CO_2_. For infection and microscopic analysis, 5 × 10^5^ cells/well were grown in 12-well cell culture-grade plates in round coverslips and incubated overnight prior to treatment. Bacterial inoculum used at a multiplicity of infection of 10 (5 × 10^6^ CFU) were incubated in the presence or absence of immune serum from AuNP-protein-LPS, AuNP-Combo2-LPS, or naive sera (final concentration of 10%) for 1 h at 37°C, with slight agitation. After incubation in the presence or absence of sera, bacteria were collected in 1 ml of fresh medium and used to infect cell culture plates containing 5 × 10^5^ cells. After 2 h of infection at 37°C with 5% CO_2_, cells were washed and fixed with 4% paraformaldehyde–PBS for 30 min. Following that step, cells were slightly permeabilized with 0.1% saponin in PBS for 10 min at room temperature. Cells were then stained with a LIVE/DEAD BacLight kit (Molecular Probes, Life Technologies) containing propidium iodide (PI) or SYTO 9 by following the manufacturer’s instructions. Cells were washed three times with PBS, fixed with 4% paraformaldehyde for 20 min, and then directly mounted using ProLong gold antifade (Molecular Probes, Life Technologies). Cells were visualized using an Olympus BX51 upright fluorescence microscope and analyzed using ImageJ software from the National Institutes of Health (51).

### Cellular immune response analysis.

Spleen cells from AuNP-Combo2-LPS-immunized mice were obtained 3 weeks after the last immunization (45). Briefly, single-cell suspensions of spleen cells from immunized and control mice (adjuvant only) were cultured in 48-well cell culture-grade plates (Corning, USA) in duplicate at 1 × 10^6^ cells/ml in RPMI 1640 (Gibco, Life Technologies) supplemented with 10% fetal calf serum (Invitrogen Life Technologies), 1 mM sodium pyruvate, 2 mM l-glutamine, 100 U of penicillin/ml, and 100 mg of streptomycin/ml (complete medium), with stimuli. Splenocyte cell suspensions were stimulated with different stimuli for 5 days, including OpcP (10 μg/ml), OpcP1 (10 μg/ml), LPS (10 μg/ml), protein plus LPS, αCD3/αCD28 magnetic antibody-coupled beads with 30 U/ml of mouse recombinant IL-2, and complete medium alone. After 5 days of incubation at 37°C in a humidified atmosphere (5% CO_2_ and 95% air), cell culture supernatants were collected and immediately stored at −80°C until further analysis. Cytokine production was analyzed using a BioPlex kit (Bio-Rad, USA) according to the manufacturer’s instructions.

### Statistical analysis.

All statistical analyses were done using GraphPad Prism software (v 6.0). *P* values of <0.05 were considered statistically significant. Quantitative data are expressed as means ± standard errors. All data were analyzed for normality before the corresponding test was run. Results of colonization, antibody, and cytokine levels were analyzed by one-way or two-way analysis of variance (ANOVA) using Tukey’s *post hoc* test or the Kruskal-Wallis *post hoc* test when data were not normally distributed. Statistical differences in survival were determined by the Kaplan-Meier method, followed by log rank test. Levels of significance compared to the adjuvant-only group: *, *P* < 0.05; **, *P* < 0.005; ***, *P* < 0.0005; ****, *P* < 0.0001.
