# Supplementary figures and images for: Multicomponent Gold-Linked Glycoconjugate Vaccine Elicits Antigen-Specific Humoral and Mixed TH1-TH17 Immunity, Correlated with Increased Protection against Burkholderia pseudomallei
Source: mBio. 2021 Jun 29;12(3):e01227-21. doi: 10.1128/mBio.01227-21 (PMC8263005; doi:10.1128/mBio.01227-21)

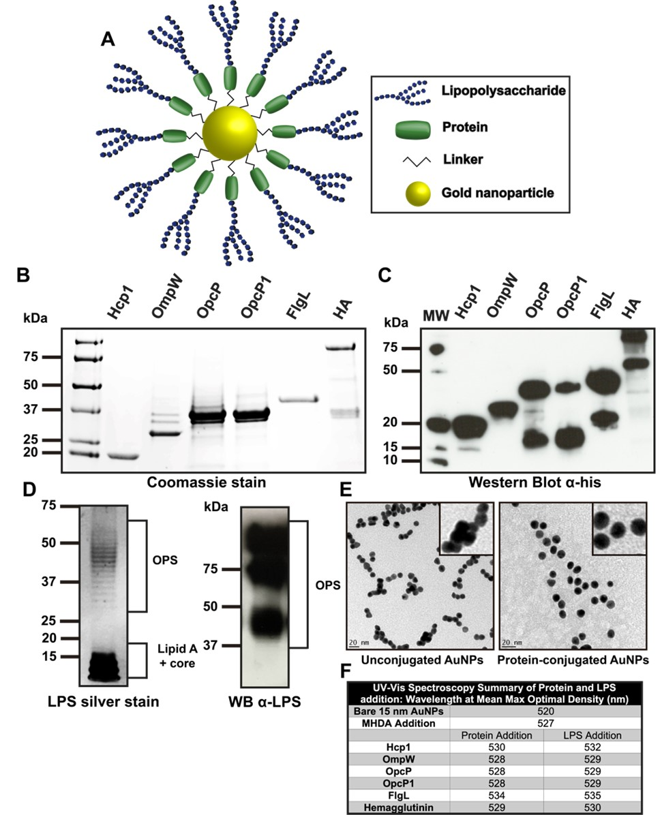

Supplement: FIG S1 [file mbio.01227-21-sf001.tif]

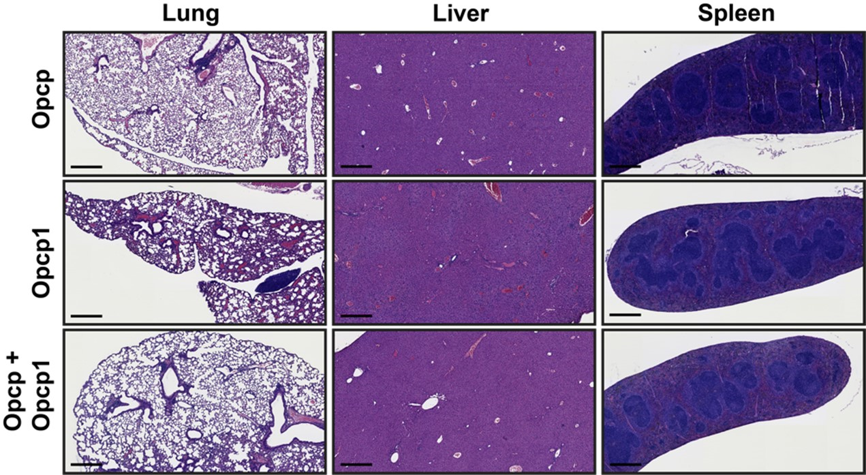

Supplement: FIG S2 [file mbio.01227-21-sf002.tif]
